# Perilymphatic Fistula After Penetrating Ear Trauma

**DOI:** 10.5811/cpcem.2019.1.37404

**Published:** 2019-03-04

**Authors:** Ashley E. Kita, Irene Kim, Gail Ishiyama, Akira Ishiyama

**Affiliations:** * David Geffen School of Medicine at the University of California Los Angeles, Department of Head and Neck Surgery, Los Angeles, California; † David Geffen School of Medicine at the University of California Los Angeles, Department of Neurology, Los Angeles, California

## Abstract

Pneumolabyrinth, defined as air within the labyrinth on high-resolution computed tomography, suggests that a perilymphatic fistula (PLF) is present. PLF describes an abnormal communication between the middle and inner ear, and can result in deafness, vertigo, and imbalance. In the setting of a penetrating injury to the temporal bone or inner ear, pneumolabyrinth should trigger prompt otolaryngology consultation and urgent surgical exploration. We describe a case in which a 49-year-old male presented with a traumatic PLF secondary to penetrating ear injury. Imaging demonstrated extensive pneumolabyrinth. Despite delay in diagnosis, expeditious surgical intervention resulted in successful preservation of inner ear function.

## INTRODUCTION

Perilymphatic fistula (PLF) refers to an abnormal communication of perilymph between the middle and inner ear through a defect in the otic capsule, and is associated with acute hearing loss, tinnitus, and vertigo. Some of the more common causes of PLF include penetrating injury (such as that of ear picks in Japan), barotrauma, middle ear surgery, and temporal bone fracture.^1^,^2^ Many reported cases of traumatic PLF deal with concurrent management of temporal bone fractures, in which case conservative strategies have been employed.^3^ As patients typically present first to the emergency department (ED), it is critical for emergency care providers to correctly identify the subgroup of patients who may require urgent surgical intervention to preserve hearing and balance. In the case of penetrating inner ear injury, high-resolution computed tomography (CT) should be carefully evaluated for air, since the presence of pneumolabyrinth confirms the diagnosis of PLF and requires urgent surgical intervention. We present the case of a man with a foreign body penetrating the inner ear who presented to the ED with spontaneous nystagmus and profound hearing loss who successfully maintained his inner ear function after immediate surgical exploration.

## CASE REPORT

After falling off a mountain bike down an incline into some brush, a 49-year-old male mountain biker presented to an outside ED with normal vitals, severe vertigo, nausea, intractable vomiting, profound hearing loss, and tinnitus. A CT was performed, which showed opacification of the ear canal, but did not comment on any abnormalities of the inner ear. The patient was transferred to our facility for further management. On examination, there was a spontaneous right-beating nystagmus and the facial nerve was intact. A tree twig was embedded in the left external auditory canal, obscuring visualization of the tympanic membrane.

**Image 1:**
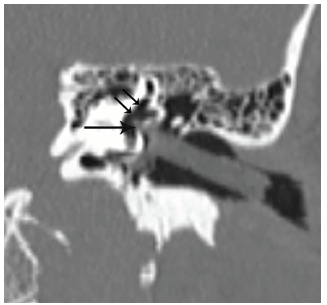
Temporal bone computed tomography reconstructed in an oblique coronal view shows a linear foreign body projecting from the external auditory canal to the oval window, with a small projection into the vestibule (arrow). Extensive intralabyrinthine air is present (double arrows).

**Image 2:**
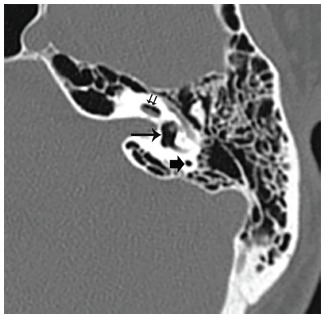
An axial cut of temporal bone computed tomography shows air in the left vestibule (long arrow), the posterior semicircular canal (short arrow), and the scala vestibuli compartment of the cochlear basal turn (short double arrows). Air was also seen in the lateral and superior semicircular canals (not shown).

Temporal bone CT demonstrated a linear foreign body projecting from the external auditory canal to the oval window, and an additional, separate small foreign body projecting into the vestibule. Presence of extensive intralabyrinthine air was detected radiographically (Image 1). On axial view, air bubbles were seen in the vestibule, posterior semicircular canal, and the scala vestibuli compartment of the cochlear basal turn, as well as in the lateral and superior semicircular canals (Image 2). Preoperative audiogram conducted at bedside revealed normal hearing on the right side and moderate-to-severe mixed hearing loss on the left.

The patient was diagnosed with a traumatic PLF with extensive pneumolabyrinth due to penetrating temporal bone injury and was taken urgently to the operating room less than one day after his inciting injury. A three- centimeter tree twig was lodged in the ear canal and found to be penetrating the tympanic membrane. Postauricular approach included mastoidectomy and intraoperative assessment of the middle ear ossicles and extent of injury. The long process of the incus was dislocated but still attached to the malleus, the stapes was deeply embedded into the vestibule, and the oval window was completely open but covered by blood clot. All penetrating foreign bodies were extracted. Temporalis fascia was used to seal the oval window and a stapes prosthesis was placed. The tympanic membrane perforation was repaired.

**CPC-EM Capsule**

What do we already know about this clinical entity?

*Trauma to the inner ear may result in hearing loss and vertigo*.

What makes this presentation of disease reportable?

*Traumatic air within the inner ear is rare, but when related to penetrating trauma it suggests a perilymphatic fistula (PLF) requiring acute operative intervention*.

What is the major learning point?

*A computed tomography should be obtained in individuals presenting with penetrating ear trauma accompanied by vertigo and hearing changes to assess for PLF*.

How might this improve emergency medicine practice?

*Clinicians should have a heightened suspicion for PLF if air is seen within the cochlea or labyrinth in a patient with hearing and balance changes*.

Postoperatively, the patient had rapid and significant improvement of his vertigo. On physical examination, there was minimal spontaneous nystagmus. A four-week postoperative audiogram revealed a mild-to-moderate mixed hearing loss in the left ear with continued improvement at six months.

## DISCUSSION

PLF describes an abnormal communication of fluid between the inner ear and the air-filled middle ear cavity.^1^ Traumatic PLF due to temporal bone fracture and traumatic PLF due to penetrating trauma of the inner ear must be distinguished, as the first can be observed with bedrest and the second requires immediate surgery. Pneumolabyrinth, or air displacing the fluid spaces of the otic capsule within the labyrinthine compartment, can confirm the presence of PLF.^4^,^5^ Physical exam findings consistent with this diagnosis include nystagmus with insufflation (positive fistula test) and dizziness induced by sound (Tullio’s phenomenon). Because this hearing loss may be conductive, sensorineural, or mixed, a tuning fork examination may be of mixed utility. As demonstrated in this case, the diagnosis of pneumolabyrinth can be easily missed.

Pneumolabyrinth may be easy to detect when it is large, but small pneumolabyrinth can be challenging to visualize on a head CT or even on temporal bone CT. In such cases, it is important to obtain coronal views of the temporal bone as air rises to the most superior aspect of the superior semicircular canals. Although the effect of air within the inner ear labyrinth is not known, animal models suggest that air bubbles disturb the propagation of the traveling wave of the basilar membrane and produce profound sensorineural hearing loss.^6^ In addition, pneumolabyrinth may cause direct irritation of the membranous labyrinth resulting in severe rotational vertigo.^4^ Of note, pneumolabyrinth in the cochlea, or pneumocochlea, is related to more severe and potentially irreversible sensorineural hearing loss than those with air confined to the vestibule.^3^,^7^

When pneumolabyrinth is present due to temporal bone fracture, conservative management with bed rest is usually recommended. Prisman et al. described three cases of pneumolabyrinth caused by temporal bone fracture in pediatric patients and reported resolution of vestibular symptoms with conservative management.^3^ The mechanism of vestibular-symptom resolution in these cases is likely due to central compensation rather than the restoration of peripheral vestibular function. In the setting of temporal bone fracture, these authors recommended surgical intervention only if the patient has cerebrospinal fluid otorrhea, progressive hearing loss, or unresolving vestibular problems.

On the contrary, pneumolabyrinth caused by penetrating temporal bone injury needs to be recognized and accurately diagnosed in the ED as best outcomes are obtained with urgent otologic consultation and immediate surgery.^8^,^9^ In this case, the presence of pneumolabyrinth was missed at an outside facility, causing a delay in diagnosis and surgical intervention. Despite this delay, the hearing and partial vestibular function were preserved. At the time of surgery, a blood clot found sealing the oval window may have prevented profound sensorineural hearing loss and permanent peripheral vestibulopathy. Given that the oval window was completely open due to subluxation of the stapes, this would have resulted in irreversible profound sensorineural hearing loss without surgery. As demonstrated in this case, the vestibular function was also partially preserved as the vestibule-ocular reflex is nearly symmetric. The diminished caloric response on the left is likely significantly overestimated secondary to tympanoplasty.

In the ED setting high-resolution CT of the temporal bone including coronal views should be obtained immediately to evaluate for pneumolabyrinth when a patient with temporal bone injury is initially assessed. Early recognition of pneumolabyrinth in this setting is crucial since urgent referral to a facility where appropriate otologic procedures can be performed could save the inner ear function.

## CONCLUSION

The long-term hearing outcomes of patients with PLF with pneumolabyrinth are generally poor, and early diagnosis and treatment are essential. Urgent physical examination, audiometry, imaging, and referral to appropriate specialists can help facilitate appropriate management. In the case of penetrating foreign body with pneumolabyrinth, urgent evaluation is critical and emergency physicians should have a high index of suspicion when patients present with findings similar to those described in this case. Our patient showed near-complete resolution of vertigo and long-term improvement in his hearing loss. These improvements in symptoms are likely attributable to the relatively brief interval to surgery and prompt repair of the PLF.
